# Hybrid Materials Based on l,d-Poly(lactic acid) and Single-Walled Carbon Nanotubes as Flexible Substrate for Organic Devices †

**DOI:** 10.3390/polym10111271

**Published:** 2018-11-15

**Authors:** Patryk Fryń, Krzysztof Artur Bogdanowicz, Natalia Górska, Jakub Rysz, Piotr Krysiak, Mateusz Marzec, Monika Marzec, Agnieszka Iwan, Adam Januszko

**Affiliations:** 1 Institute of Physics, Jagiellonian University, Prof. S. Lojasiewicza 11, 30-348 Krakow, Poland; patryk.fryn@doctoral.uj.edu.pl (P.F.); jakub.rysz@uj.edu.pl (J.R.); 2 Military Institute of Engineer Technology, Obornicka 136 Str., 50-961 Wroclaw, Poland; bogdanowicz@witi.wroc.pl (K.A.B.); krysiak@witi.wroc.pl (P.K.); 3 Faculty of Chemistry, Jagiellonian University, Gronostajowa 2, 30-387 Kraków, Poland; gorska@chemia.uj.edu.pl; 4 Academic Centre for Materials and Nanotechnology, AGH University of Science and Technology, Av. Mickiewicza 30, 30-059 Krakow, Poland; marzecm@agh.edu.pl; 5 Faculty of Security and Safety Research, General Tadeusz Kosciuszko Military University of Land Forces, Wroclaw, MULF Wroclaw, Czajkowskiego 109 Str., 51-147 Wroclaw, Poland; adam.januszko@awl.edu.pl

**Keywords:** l,d-poly(lactic acid), single wall carbon nanotubes, thermographic camera, hybrid materials, flexible substrate

## Abstract

We report on the application of l,d-poly(lactic acid) (l,d-PLA) with dispersed Single-Walled Carbon Nanotubes (SWCN) as a flexible translucent electrode for organic devices. We used commercially available nanotubes in various weight ratios from 0 to 8% dispersed in chloroform polymeric solution by ultrasonication and were drop cast. The created hybrid materials were investigated by differential scanning calorimetry to determine the influence of SWCN content on the thermal behavior, while polarizing optical microscope was used to find the effect of mechanical deformations on the textures. Drop-cast films were studied by optical transmittance, conductivity, dielectric properties and by thermal imaging under applied potential. Thermal imaging provided evidence of visible voltage-activated conduction. Simple mechanical deformation such as bending with stretching at edge to ca. 90 and elongation test were performed. Moreover, interactions between l,d-poly(lactic acid) and SWCN were investigated by FT-IR and NMR spectroscopy. Finally, we can conclude that the thermographic examination of created films permits fast, simple and inexpensive localization of defects on the surface of l,d-PLA:SWCN film, together with the electrical properties of the films.

## 1. Introduction

Organic devices, such as organic solar cells, for example, are mainly constructed on a glass substrate [1,2,3,4,5,6,7,8,9,10,11,12,13,14,15,16]. However, organic solar cells on flexible substrates have also been investigated for potential use in our life [17,18,19,20,21,22,23,24,25,26,27,28,29,30,31,32]. Up to now, only few reports have been dedicated to flexible organic solar cells, because of the still low power conversion efficiency (PCE) value in comparison with organic solar cells built on glass substrates [17,18,19,20,21,22,33]. Our literature search showed that, as a substrate in organic (polymer) solar cells, predominantly polyethyleneterephthalate (PET) has been tested, and the highest PCE value was observed to be approximately 4.2% [16,34]. Moreover, other polymers such as polyethylene naphthalene [23,25] and polyethersulfone [31] have been tested as a flexible substrate in polymer solar cells with ITO or graphene as an electrode, giving similar results.

In our last study we investigated Ecoflex^®^ as a substrate covered with graphene oxide for application in organic solar cells [35], and this inspired us to think about the possibility of applying poly(lactic acid) (PLA), widely known as biodegradable material [36], in organic devices. It is well known that due to the chiral nature of lactic acid, several distinct forms of polylactide exist, such as l-PLA, d-PLA, and l,d-PLA which exhibit different properties, especially with regard to melting temperature and crystallinity [37,38]. Strange et al. [39] investigated l-PLA in polymer solar cells as a substrate loaded with nanoclay in an attempt to improve the thermal properties and reduce permeation of water and oxygen. As an alternative, the usage of a new biodegradable polymer electrolyte was proposed for a Dye Sensitized Solar Cell (DSSC); a biopolymer containing about 2–3% starch (20 to 25% amylose and 75 to 80% amylopectin) called Arrowroot (*Maranta arundinacea*) was applied [40]. On the other hand, Zhou et al. [41] investigated recyclable organic solar cells on cellulose nanocrystal substrates. 

However, with respect to biodegradable, flexible organic solar cells, not only is an adequate substrate required, but also nanomaterials that provide suitable electrical properties, such as work function or resistivity. Presently, instead of indium tin oxide (ITO) graphene, Ag, or carbon nanotubes are mainly proposed as electrode [5]. It is well known, that ITO on flexible substrates like PET and polyimide possesses good optical and electrical properties. However such ITO possesses disadvantages, such as: (i) the limited annual production of indium, as a rare metal, causes an increase in ITO prices; (ii) ITO is brittle and cannot withstand mechanical deformation; and (iii) degradation of ITO in organic solar cells is caused by PEDOT:PSS used as hole transporting layer, eliminating its wide, cheap and long-term application. Therefore, finding substitutes to ITO is of great significance in organic devices [42].

Both single-walled carbon nanotubes (SWCN) and multi-walled carbon nanotubes (MWCN) have been widely investigated since 2004 as transparent conductive films in organic devices, and they have shown promising applicability in optoelectronic devices and as solar cells, organic light-emitting diodes (OLEDs) and touch panels [42,43]. 

Carbon nanotubes (CN), among numerous diverse applications, can be used as an electrode in organic solar cells on flexible substrates; exemplary configurations of flexible substrate and electrode can be named: PET/CN, or PET/poly(aniline):CN [42]. Moreover, Calogero et al. [44] proposed SWCN deposition on stainless-steel sheet substrates as novel counter electrodes for ruthenium polypyridine-based DSSC. 

An interesting application of CN was proposed by Husmann and Zarbin, in which multifunctional carbon nanotubes/ruthenium purple thin films were tested as sensors and electrochromic materials [45]. 

Additionally, the creation of a suitable composition of polymers, as well as the interactions between both compounds, is not trivial for the excellent applicability of carbon nanotubes. For example, hydrogen-bonded metallo-supramolecular polymers based on ruthenium or iron complexes for the selective extraction of SWCN were investigated by Oka et al. [46].

Noteworthy work was done by Ma et al. [47] in which a novel route was proposed to fabricate entirely bio-based conductive nanocomposites with a low percolation threshold of conductivity and enhanced crystallization and mechanical performance. To achieve this goal, the authors proposed using poly(d-lactide)/poly(hydroxyalkanoate) (PDLA/PHA) nanocomposites with multiwalled carbon nanotubes (MWCNTs) and MWCNTs-*g*-PLLA (prepared via ring opening polymerization of l-lactide with grafting degree of 17%).

Instead of carbon nanotubes, graphene has also been applied in PLA compositions. For example, Lei et al. [48] prepared conductive PLA with poly(methyl methacrylate) (PMMA) functionalized graphene by admicellar polymerization. Moreover, Zhang et al. [49] investigated a series of transparent PLA nanocomposite films incorporated with fully exfoliated octadecylamine-functionalized graphene as an effective nanofiller. On the other hand, Cui et al. [50] studied highly heat-resistant PLA-based foam with low foam density and excellent electromagnetic interference shielding performance fabricated using the nonsolvent-induced phase-separation and freeze-drying method.

All scientists agree that taking into account the possible applications of flexible organic devices in our lives is still a challenge. One of the challenges is the construction of efficient devices with long-term stability. To solve this problem, each layer in the device should be optimized. On the other hand, considering organic devices as ecologically friendly devices according to Green Chemistry concepts, new flexible and translucent substrates based on biodegradable components are necessary. 

Therefore, in this study, we propose hybrid, biodegradable thin films based on Single-Wall Carbon Nanotubes (SWCN) and l,d-poly(lactic acid) (PLA) as biodegradable polymer. We report the influence of the amount of SWCN and mechanical deformation on the electrical and thermal behavior of l,d-PLA. We paid a lot of attention to investigating l,d-PLA/SWCN interactions using FT-IR and NMR methods, with a view towards applications in organic biodegradable devices. 

It is known that organic devices could work for industrial applications, such as solar panel. One of the main problems in solar panels is the presence of various mechanical and structural defects that cause a decrease in power generation. To analyze defects in solar cells, various techniques have been used; however, thermal imaging is a fast and simple method for identifying and locating defects. Therefore, one of the main goals of this work was to analyze defects in the produced flexible l,d-PLA:SWCN films via thermal imaging. 

## 2. Experimental

### 2.1. Materials

**Figure 1:**
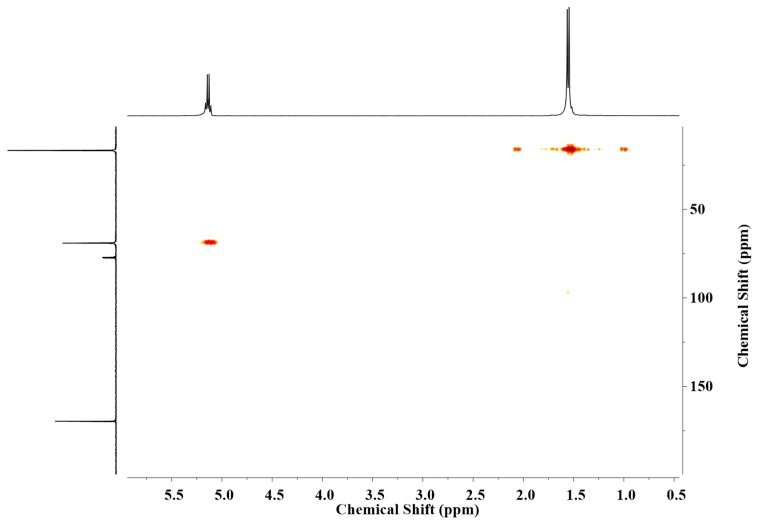
^1^H–^13^C NMR (HMQC) spectra for l,d-PLA.

The chemicals and reagents were used as received from Sigma-Aldrich (St. Louis, MO, USA). The Single-Walled Carbon Nanotubes (SWCN, Sigma-Aldrich, St. Louis, MO, USA) used have average diameter 0.84 nm, median length 1 µm, ≥95% carbon basis (≥99% as carbon nanotubes). l,d-PLA was received from GALACTIC. (Brussels, Belgium). Chloroform 98.5% PURE P.A.—BASIC (POCH S.A).

l,d-PLA: ^1^H NMR (400 MHz, CDCl_3_), δ [ppm]: 5.1 (m, 1H, –(C=O)–CH<), 1.5 (m, 3H, >CH–CH_3_). ^13^C NMR (100 MHz, CDCl_3_), δ [ppm]: 169.7 (aliphatic carboxyl, –(C=O)–), 69.1 (methyline, –(C=O)–CH<), 16.7 (methyl, >CH–CH_3_). The l,d-PLA were characterized by ^1^H–^13^C HMQC (see Figure 1). Approximate number of repetitive units = 101. Approximate average molecular mass = 7274 g/mol.

FT-IR: 2995 m ν_as_(CH_3_), 2965 sh ν(CH_3_), 2944 m νs(CH_3_), 2879 w, 2861 w, 1748 s ν(C=O), 1722 sh, 1453 m δ_as_(CH_3_), 1382 m δ_s_(CH_3_), 1360 m δ_s_(CH_3_), 1302 w, br δ(CH), 1268 m ν(C–O), 1209 sh, 1180 s ν_s_(C–O–C), 1127 s ν(C–O), 1081 s ν_as_(C–O–C), 1043 s ν(C–O), 1020 sh, 956 w, 919 w, 896 w, 869 m ν(C–C), 847 sh, 754 m ν(C–C), 735 sh, 706 br, 669, 394 m cm^−1^ (s–strong, m–medium, w–weak, br–broad, sh–shoulder).

#### Preparation of l,d-PLA:SWCN Hybrid Materials

To prepare the mixture of l,d-PLA with SWCN, the appropriate amounts of l,d-PLA and SWCN were mixed in chloroform to obtain a solution. The solution was stirred on the magnetic stirrer for 1 h, and then sonification (60%) was performed for different durations depending on the concentration: l,d-PLA/0.01% SWCN, l,d-PLA/0.1% SWCN and l,d-PLA/1% SWCN (80 cycles) while l,d-PLA/5% SWCN and l,d-PLA/8% SWCN (40 cycles); 1 cycle = 30 s sonification + 30 s interruption. The l,d-PLA mixed with chloroform was only stirred on the magnetic stirrer for 1 h, without sonification after that. All the solutions were then poured into 8 cm glass Petri dishes and kept at room temperature for 12 h to evaporate the chloroform. After that time, the samples had the form of thin flexible layers (8 cm diameter), and easily detached from the bottom of the Petri dishes. The thickness of the samples was measured by the profilometric method.

### 2.2. Characterization Methods

DSC measurements were done by using differential scanning calorimeter Perkin Elmer Diamond 8000. Samples of ca. 10 mg were placed in aluminum crucibles and firmly closed with a press. The measurements were carried out both for heating and cooling at a rate equal to 5 °C/min. 

Texture observations were done using a Nikon Eclipse LV100 POL polarizing microscope (NIKON Inc., Tokyo, Japan). The images were registered in transmitted light (90° crossed polarized) by a computer-controlled camera at room temperature. The samples were placed between two glass plates. 

Mechanical deformation was carried out by bending and stretching samples on an edge of ca. 90°. The procedure was repeated twenty times for each side of the sample. 

Tensile mechanical properties were measured using a testing machine Instron 33R4469 (Instron, Norwood, MA, USA) with a load cell of 5 kN with software Bluehill 3.0. Samples were in a form of thin strips and uniform width of 10 mm. Samples were tested at room temperature at a crosshead speed of 10 mm/min.

Resistivity R was measured at twenty different places on the samples using the four point method, by using a Source Meter Keithley 2400 (KEITHLEY, Cleveland, OH, USA). 

**Figure 2:**
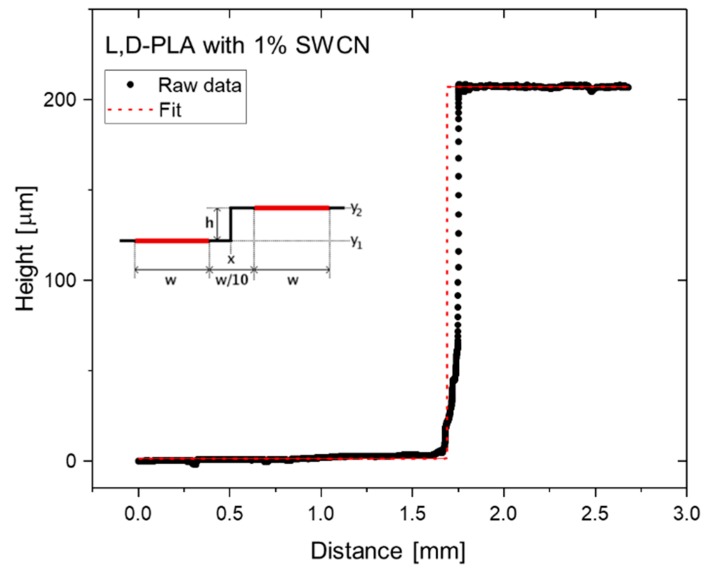
Topography profile (dots) obtained from profilometer measurements used for the determination of l,d-PLA with the 1% SWCN sample thickness by fitting edge function (dashed line): y_1_ and y_2_ are baselines, and h is the resultant thickness.

The thickness of the analyzed samples was determined using DektakXT (Bruker Co., Billerica, MA, USA) Stylus profilometer working in N-Lite mode, which ensures very low stylus load (radius 2 µm, load 0.3 mg) on the sample surface, ensuring non-destructive analysis. The edge of each sample was measured, and the edge type function was fitted to the obtained data. A schematic illustration of the acquired data and function fitted for l,d-PLA with 1% SWCN is depicted in Figure 2. The sample thickness for SWCN concentrations equal to 0.1%, 1%, 5% and 8% are presented in Table 1. The remaining thicknesses of the samples were equal to 29.1 and 282.1 µm for l,d-PLA from chloroform and the 0.01% SWCN mixture, respectively.

Dielectric measurements at room temperature were performed by using Novocontrol Alpha Spectrometer in the frequency range 0.1 Hz–10 MHz at a measuring oscillation voltage of 0.5 V. The samples, in the form of circle 2 cm in diameter, were placed between the two electrodes.

Thermal behavior was observed using a thermographic camera (VIGOcam v50, VIGO System S.A, Ozarów Mazowiecki, Poland) while applying voltage bias of between 0 and 32 V using a conventional DC source (Regulated Power Supply PWR 400L (KIKUSUI)).

NMR results were recorded on a Jeol ECZ-400 S 400 MHz with a delay time of 5 s. Deuterated chloroform (CDCl_3_) was used as solvent. Measurements were carried out at room temperature on 10–15% (*w*/*v*) sample solutions. Heteronuclear multiple quantum correlation (HMQC) experiments were performed according to standard procedures.

ATR FT-IR spectra were collected using an ALPHA Bruker spectrometer equipped with a 1-reflection ATR diamond crystal (Bruker Co., Billerica, MA, USA). Spectra were collected at room temperature in the range of 4000–500 cm^−1^, with a spectral resolution of 4 cm^−1^ and at 32 scans per spectrum. All spectrum processing was performed using Bruker OPUS software (Version 7.0).

The optical properties of polymer samples modified with carbon nanotubes in the range from 400 nm to 900 nm were characterized using a home-built system based on the spectrometer USB2000 with linear CCD detector (OceanOptics Ltd., Largo, FL, USA) controlled by SpectralSuite software. Light from a halogen lamp directed on the studied film and the transmitted signal were registered by the spectrometer, and the absorbance spectrum was calculated based on the previously stored reference spectrum of the lamp. The reflection signal was collected using a fiberoptic reflection probe (OcenOptics, Largo, FL, USA). 

## 3. Results and Discussion 

**Figure 3:**
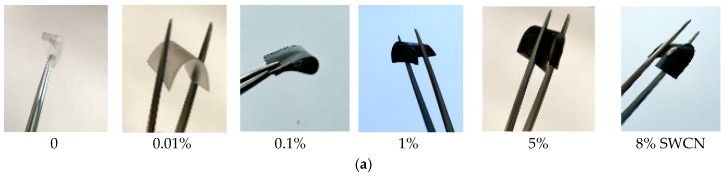

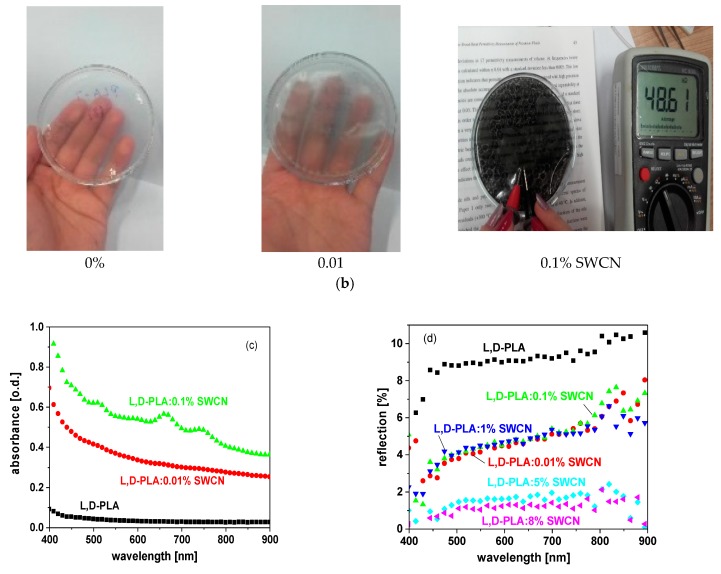
(**a**) Created l,d-PLA films without and with SWCN content of 0.01%, 0.1%, 1%, 5%, 8%, (**b**) translucence of l,d-PLA films without and with SWCN content of 0.01%, and 0.1%, (**c**) absorbance and (**d**) reflection UV-Vis spectra.

The l,d-PLA was mixed with SWCN in different ratios and drop-cast on glass substrate to create a flexible layer after chloroform evaporation. In Figure 3a,b photos of the created films are presented to show their transparency and flexibility, along with the increase in the amount of SWCN. Figure 3c presents the absorbance spectra of pure l,d-PLA and two films admixed with 0.01% and 0.1% of SWCN. Unfortunately, for high concentrations, no light is transmitted through the film. The reflection spectra of all prepared samples are presented in Figure 3d. The absorbance of the polymer films admixed with single-walled carbon nanotubes increases rapidly with their concentration so that films containing 1% SWCN become non-transparent. The shape of the absorption spectrum of the film with 0.1% of SWCN corresponds to the absorption spectrum of SWCN in solution. The most pronounced maximum, at c.a. 640 nm, corresponds to absorption of SWCN with chirality (7,6). The other two absorption bands at c.a. 720 and 500 nm correspond to SWCN with chirality (8,7) and (7,3), respectively. The reflectance of the films declines with SWCN content from less than 10% for polymer film to less than 2 for film admixed with 8% SWCN, which also indicates the high absorption of light into the film.

### 3.1. DSC Measurements and Texture Observations before and after Mechanical Deformation

**Figure 4:**
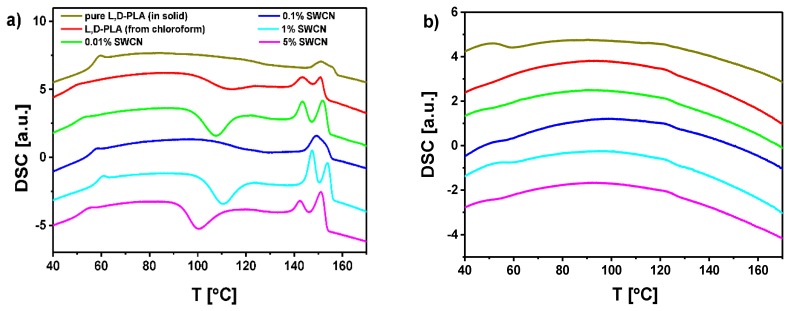
DSC curves registered for l,d-PLA and its mixtures with SWCN on heating (**a**) and cooling (**b**) at a rate of 5 °C/min.

l,d-PLA and its mixtures with SWCN (different concentrations) were studied using the DSC method to determine the influence of content of SWCN on the thermal behavior. Three cycles of heating and cooling were done for each sample, and the obtained results were repeatable. The DSC curves registered upon heating and cooling at a rate of 5 °C/min are presented in Figure 4a,b, respectively. DSC measurements show that the SWCN content has no significant influence on the phase transitions. According to the literature [51], the peak registered upon heating at ca. 150 °C is connected with melting (Δ*H* ca. 19–20 J/g, calculated as the sum of two peaks registered close to each other), while the peak at around 55 °C was registered at both heating and cooling as a glass-transition (Δ*H* ca. 1–1.5 J/g). In turn, the exothermic anomaly visible at ca. 100 °C during heating is related to chloroform (solvent used during preparation of the mixtures). In this case, enthalpy changes depending strongly on concentration of SWCN, and is equal to −10.6 J/g (l,d-PLA), −21.1 J/g (0.01% SWCN), −7.0 J/g (0.1% SWCN), −17.6 J/g (1% SWCN), −18.0 J/g (5% SWCN) and −12.8 (8% SWCN), which means that chloroform still exists in different volumes in the structure of the obtained layers. This peak was not observed for pure l,d-PLA as a solid (Figure 4a) or for l,d-PLA prepared from the solution in methylene chloride, while it appeared for l,d-PLA prepared from the solution in chloroform (Figure 4a).

**Figure 5:**
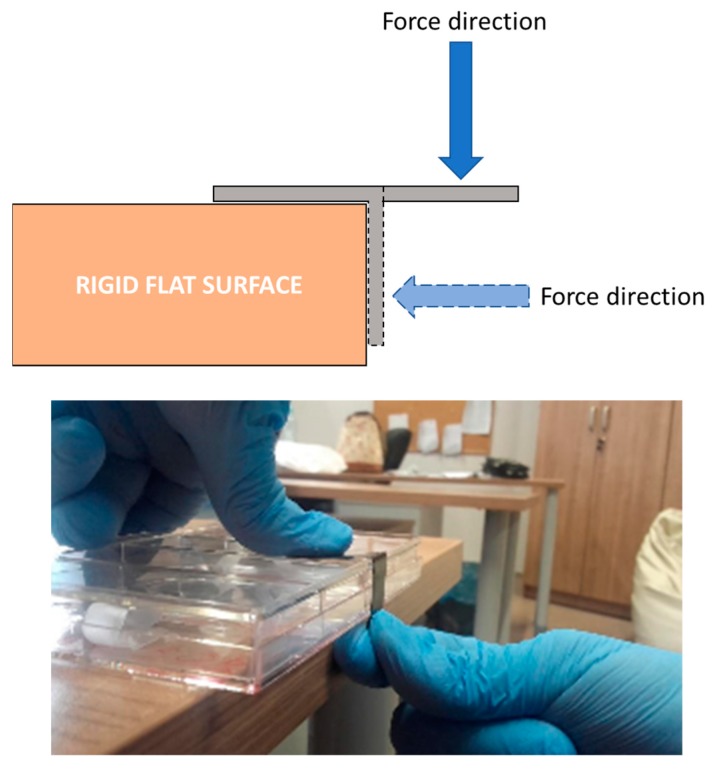
Schematic representation of simple preliminary mechanical deformation procedure together with photo of deformation of l,d-PLA film with SWCN.

To define the possible applicability of l,d-PLA in organic devices, the obtained hybrid films were investigated by optical microscopy and thermographic camera before and after mechanical deformation. To simulate mechanical damage, samples were bent and stretched 20 times, where half of the sample was placed and held on the edge of a rigid flat surface, and the other half was suspended in air. Next, a force was used to bend the sample to the side of the support, as depicted in Figure 5.

**Figure 6:**
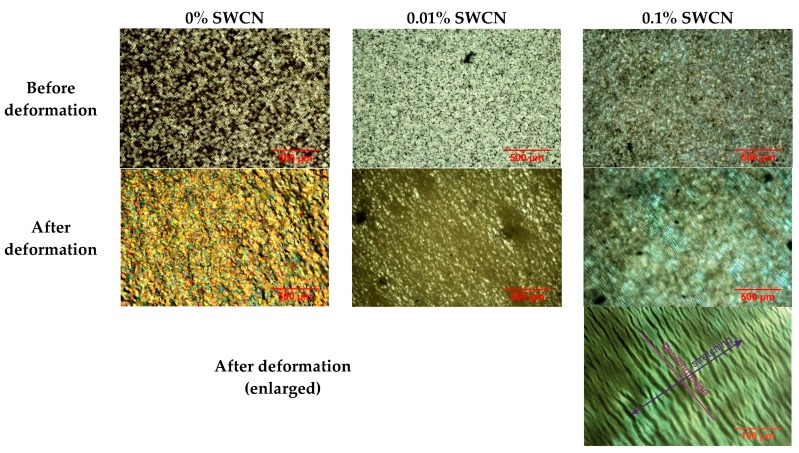
Optical texture for l,d-PLA and its mixtures with SWCN before and after mechanical deformations; room temperature, crossed polarizers (90°).

Optical textures under polarizing microscope (90° crossed polarizers) in transmitted light were analyzed for l,d-PLA and hybrid samples with SWCN concentrations equal to 0.01% and 0.1% before and after mechanical deformations, whereas samples with concentration of 1%, 5% and 8% SWCN were opaque for white light—they are completely black. Textures registered at room temperature for pure l,d-PLA and the three mixtures before and after mechanical deformations are presented in Figure 6. The effect of mechanical deformation is clearly visible for all studied samples. For pure l,d-PLA after deformation, the structure and texture, as well as the color, were changed, which means that internal stress had occurred. For the sample with a concentration of 0.1% SWCN, the color did not change, but numerous micro-cracks appeared, which is clearly visible on the enlarged texture in Figure 6. The orientation of the micro-cracks is parallel to the edge on which the bending and stretching took place. On the other hand, for the SWCN concentration of 0.01%, the changes in texture are between that for pure l,d-PLA and its mixture with 0.1% SWCN content (a slight change in color and micro-cracks appear). When increasing the concentration of SWCN in the sample, the amount of micro-cracks increases, internal stress diminishes (change in the texture color vanishes) and stretching was able to damage the sample. This suggests that the presence of SWCN stiffens the structure of l,d-PLA.

### 3.2. Conductivity of Hybrid Films

Based on the measured resistivity (*R*) and thickness (*d*) of the samples, the conductivity (σ) was calculated before and after deformation of the samples [52]:

(1) $$\sigma ==\frac{1}{R}\frac{ln2}{\pi d}$$

All measured and calculated results are gathered in Table 1. Mixtures with concentrations of SWCN lower than 0.1% are dielectrics (as shown below), and they are omitted from Table 1. It is seen that the conductivity is the lowest for the composite with a SWCN concentration of 0.1%, and increases when increasing the SWCN concentration. Moreover, the influence of deformation on conductivity is that conductivity value is lowered after deformation.

**Figure 7:**
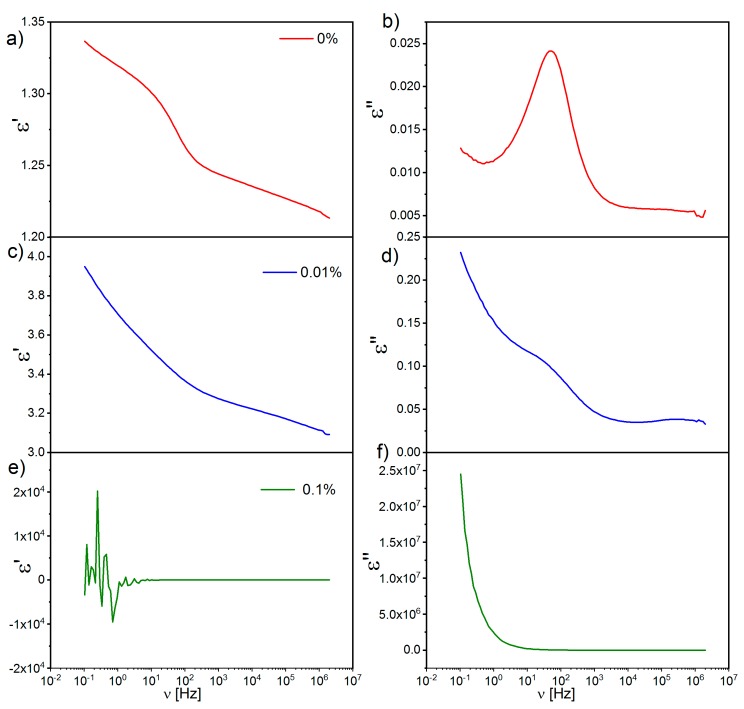
Dielectric dispersion (**a**) and absorption (**b**) registered for pure l,d-PLA, dielectric dispersion (**c**) and absorption (**d**) registered for mixture with SWCN content of 0.01%, dielectric dispersion (**e**) and absorption (**f**) registered for mixture with SWCN content of 0.1%; room temperature.

Measurements of dielectric permittivity were done for three samples: pure l,d-PLA and l,d-PLA with SWCN concentrations equal to 0.01% and 0.1%, because mixtures with higher concentrations are good conductors (see Table 1). The dielectric spectra registered at room temperature for these three thin films are presented in Figure 7. As can be seen, the amount of SWCN has an influence on the dielectric spectra. While layers prepared with l,d-PLA without SWCN are dielectric with a very clearly visible relaxation at 100 Hz, the layer with 0.1% SWCN is conductive, and relaxation is not visible. In turn, for the layer with a SWCN concentration of 0.01%, a weak relaxation is still visible. Moreover, dielectric dispersion ε’ at the low-frequency limit for the composite with a SWCN concentration of 0.01% (Figure 7c) is noticeably higher than for the pure l,d-PLA sample (Figure 7a). On the other hand, for the mixture with a SWCN concentration of 0.1%, the dielectric dispersion is characteristic of conductive materials (Figure 7e).

### 3.3. Thermal Images of l,d-PLA with SWCN before and after Bending

**Figure 8:**
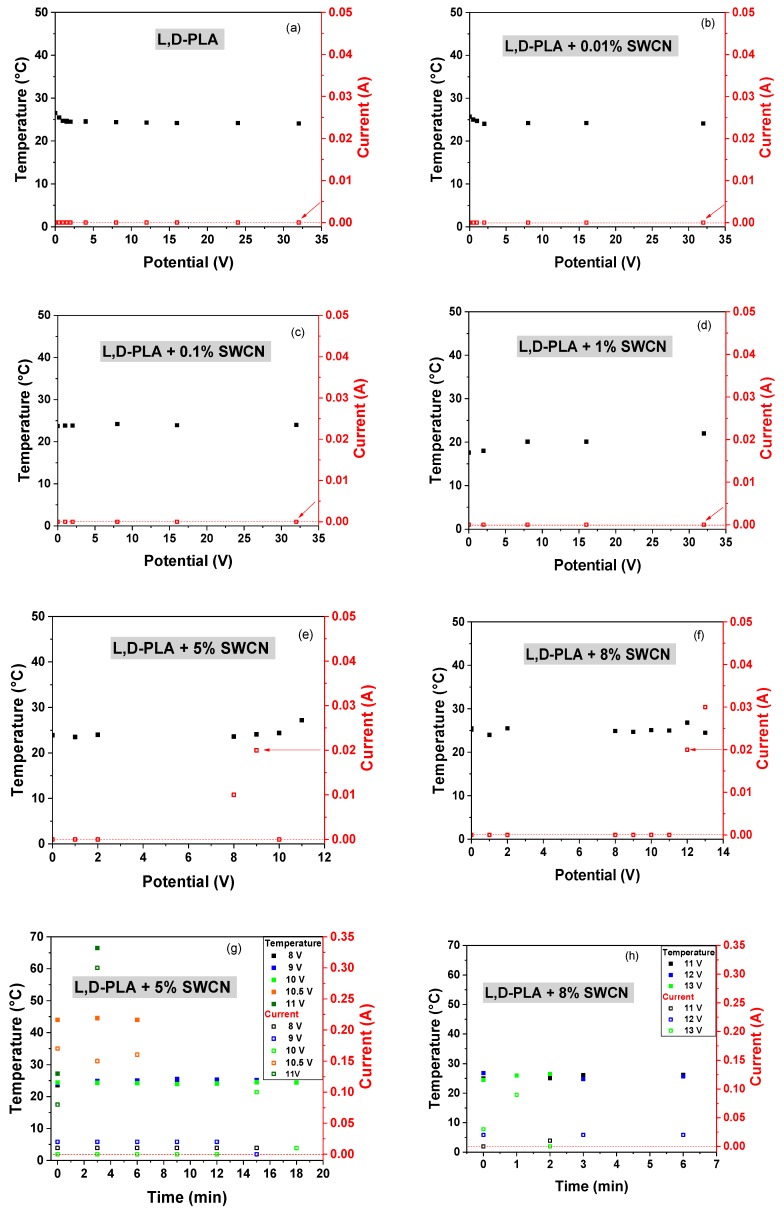
The correlation of temperature and current at different applied potentials for the film l,d-PLA (**a**) and films based on l,d-PLA containing 0.01% (**b**), 0.1% (**c**), 1% (**d**), 5% (**e**) and 8% (**f**) SWCN, together with the correlation of temperature and current over time for films based on l,d-PLA containing 5% (**g**) and 8% (**h**) SWCN; before mechanical deformation.

Thermal images of the l,d-PLA self-supported thin films containing different amounts of SWCN were acquired with a thermographic camera at different bias potentials, to observe IR emission due to current flux (i.e., Joule heating) and to detect possible current shunts. Figure 8 presents the correlation of temperature and current at different applied potentials for films based on pure and hybrid l,d-PLA. The samples of l,d-PLA with 0.01%, 0.1% and 1% SWCN did not reveal any temperature change with applied potential in the range from 0 to 32 V, and no current was observe above 0.01 A. These findings are in good accordance with electrical experiments; the samples with lower SWCN content showed higher values of ohmic resistance among all the tested materials.

On the other hand, samples containing 5% and 8% of SWCN (see Figure 8e,f) are much better conducting materials due to higher content of carbon additives and demonstrated an increase of temperature when applying different bias potentials. The analyses of the IR images, represented as temperature and current correlation vs. applied potential, are presented in Figure 8. An increase in the temperature was observed at 9 and 12 V for contents of 5% SWCN and 8% SWCN, respectively. Only one peculiar behavior was observed for films with 5% SWCN: when increasing the potential from 9 to 10 V, the current suddenly drops below the level of 0.01 A, which might suggest a local polarization and local charge accumulation on the surface of the insulator domains [53], which in this case would be related to the presence of l,d-PLA.

Regarding the time-dependent measurements at fixed values of applied potentials, the following tendency was observed: for currents at level of 0.01 A, no increase of temperature was observed; whereas for currents above 0.1 A, there was an increase in the temperature of the sample, as can be seen in Figure 8g,h.

**Figure 9:**
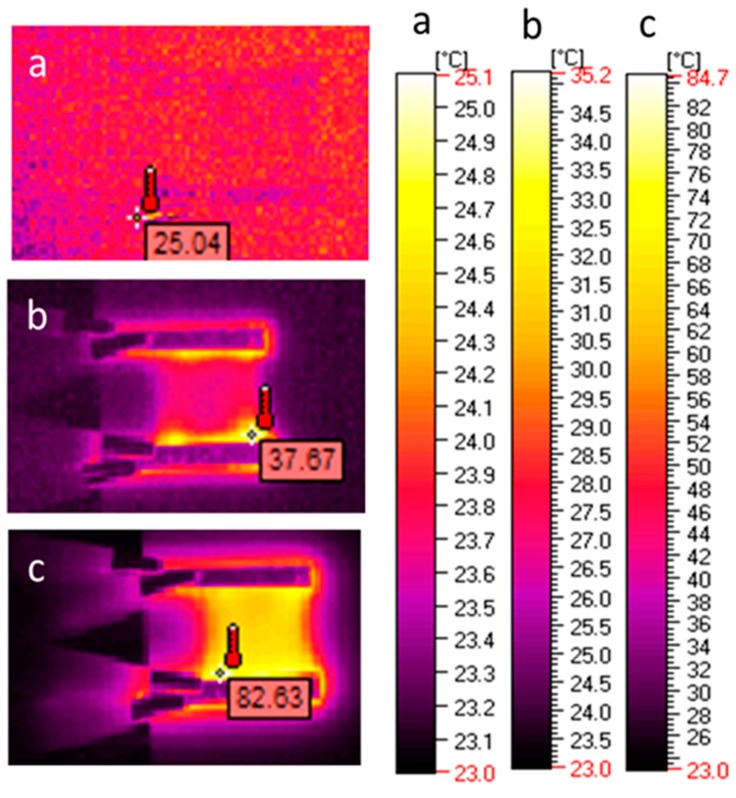
IR mages obtained for l,d-PLA with 5% SWCN under different potential value: 2 V, 0 A (**a**); 11 V, 0.08 A (**b**); and 11 V, 0.3 A (**c**).

Analyzing the images with regard to the temperature distribution on the example of l,d-PLA with 5% SWCN (Figure 9), it can be observed that the zone of increased temperature appeared at the side of the alumina contactors, at the interface between the electric contactors and the tested film (Figure 9b); when the current was equal to 0.08 A, the temperature increased from 24 to 27.2 °C. When the current reached a value of 0.3 A, the sample temperature also increased to 66.5 °C (Figure 9c).

**Figure 10:**
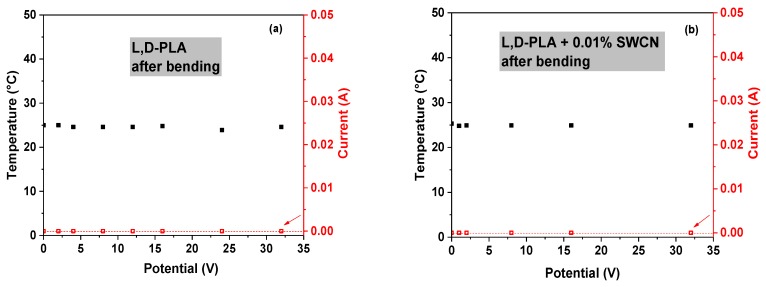

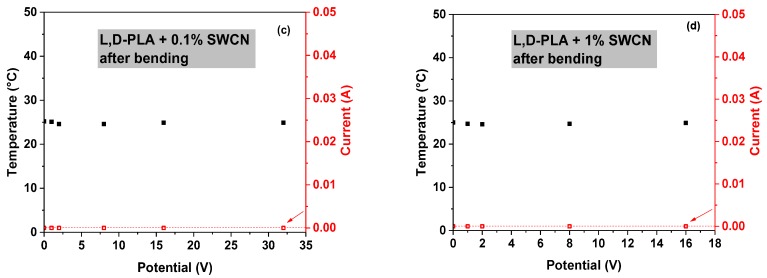
The correlation of temperature and current at different applied potentials for l,d-PLA film (**a**), and films based on l,d-PLA containing 0.01% (**b**), 0.1% (**c**) and 1% (**d**) SWCN; after mechanical deformation.

In the following step, analysis of the film after mechanical deformation was performed. The results, showing the correlation of the temperature and current with applied potential for pure l,d-PLA in the samples containing 0.01%, 0.1% and 1% SWCN in the polymeric matrix are shown in Figure 10. These samples did not reveal any temperature change in the range of applied potential from 0 to 32 V, and no current flow was observed above 0.01 A. Therefore, materials before and after mechanical deformation present similar properties.

**Figure 11:**
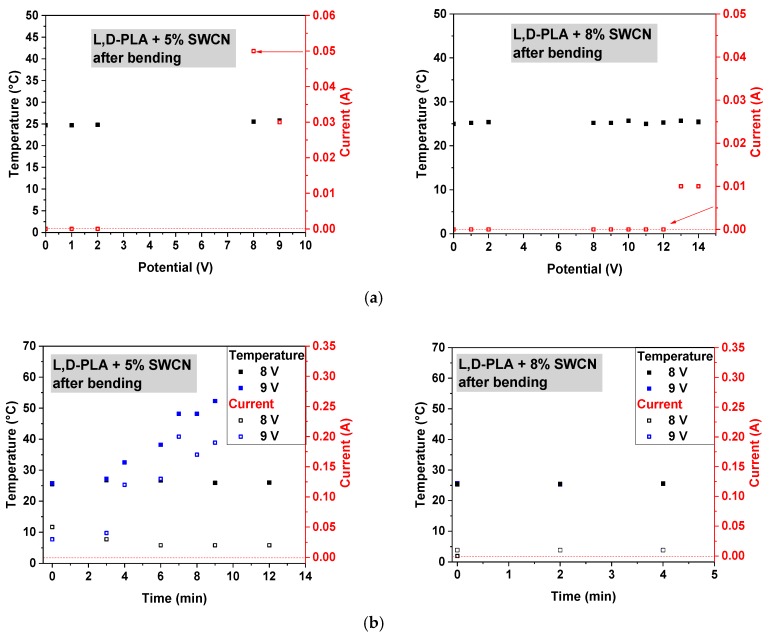
The correlation of temperature and current at different applied potentials (**a**) and the correlation of temperature and current over time (**b**) for films based on l,d-PLA containing 5% and 8% SWCN; after mechanical deformation.

Similar to previous findings, only samples containing 5% and 8% SWCN did not lose their conducting properties after mechanical deformation; however, some deterioration in the performance was noticed (Figure 11a). Increasing the potential to 14 V, the maximum observed currents were on levels up to 0.05 and 0.01 A for 5% and 8%, respectively, which were similar to the results before deformation. Moreover, no temperature change of the sample was observed for 8% SWCN content.

Considering the application of constant potential over time, only in the case of 5% addition of SWCN was an increase in the temperature observed, along with an increase in current flux, as can be seen in Figure 11b.

**Figure 12:**
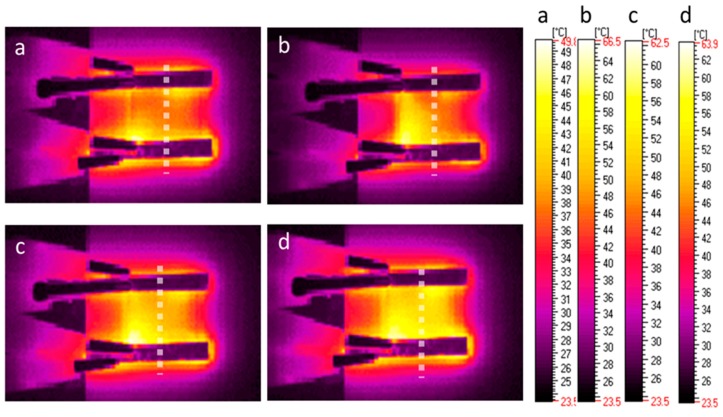
IR images obtained for l,d-PLA with 5% SWCN under different potential values: 9 V, 0.13 A, 6 min (**a**); 9 V, 0.2 A, 7 min (**b**); 9 V, 0.17 A, 8 min (**c**); and 9 V, 0.02 A, 9 min (**d**); after mechanical deformation.

The analysis of the homogeneity of the temperature distribution on the example of l,d-PLA with 5% SWCN after deformation (Figure 12), showed the differentiation of the heating zone. The line of the bending was located in the middle of the sample, perpendicular to the aluminum contactors. As previously mentioned, heating starts from the interface between the Al contactors and the sample (Figure 12a), and expands only on the top side of sample, where the electric circuit is the shortest, marking the bending line (Figure 12b). However over time, the temperature increases and expands over the whole sample (Figure 12c,d).

### 3.4. Mechanical Properties of l,d-PLA with SWCN

**Figure 13:**
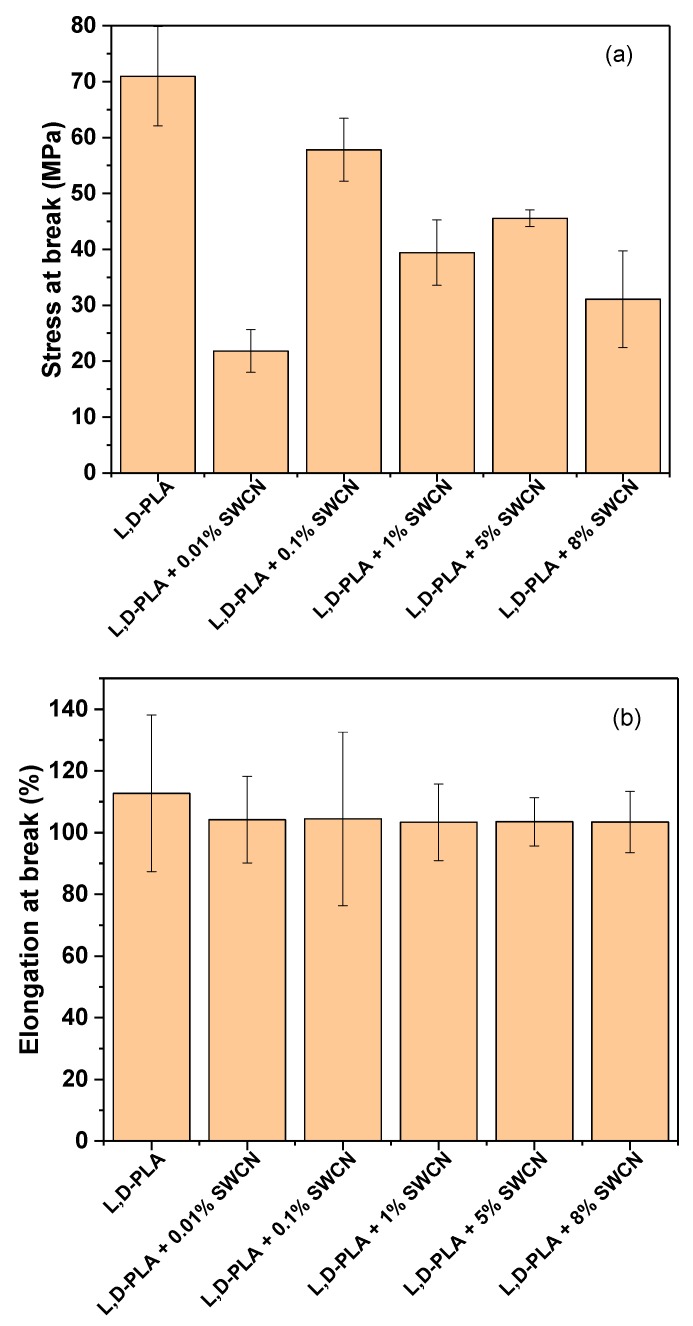
Stress at break (**a**) and elongation at break (**b**) of l,d-PLA with SWCN.

Mechanical properties were studied on prepared thin films to elucidate the influence of carbon nanotubes on mechanical resistance. All results are listed in Table 2. As can be seen from the relation of stress at break for different samples (Figure 13a), a small amount of SWCN such as 0.01% has a significant impact on material properties, resulting in an almost 3-fold decrease in stress resistance during elongation compared to pure l,d-PLA. For other SWCN concentrations, the stress values are lower than for pure polymers but do not surpass the value for l,d-PLA with 0.01% SWCN. This finding could be related to the formation of microdomains; hence, the interaction with the matrix is limited due to the possibility of weakening the structure [54].

Also, a tendency can be observed for other samples: with SWCN content starting from 0.1% and increasing to 8%, the stress values declined gradually from approximately 60 to 31 MPa, taking into account the experimental error. This can be explained by the loss of plasticity with increased content of SWCN.

Taking into account elongation at break (Figure 13b), the materials showed similar results, regardless of the specific content of SWCN in the l,d,-PLA matrix, giving an increase of only up to 4% of the original length.

### 3.5. Changes in the Chemical Structures of l,d-PLA by SWCN Studied by FT-IR

**Figure 14:**
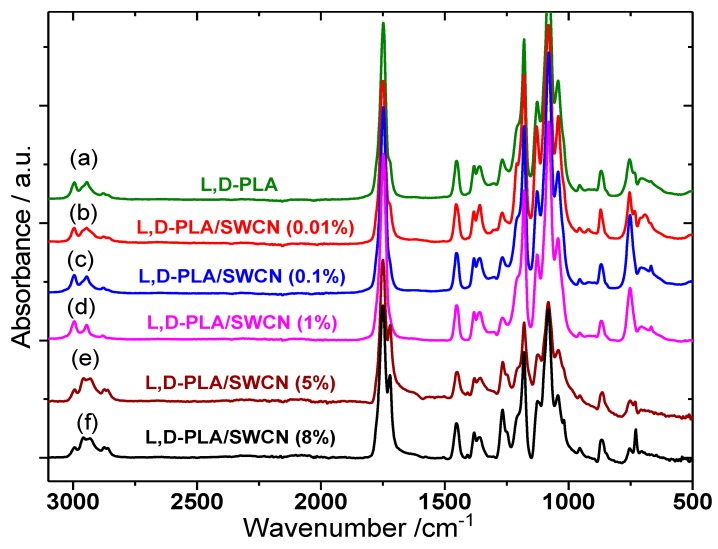
FT-IR spectra of l,d-PLA and l,d-PLA/SWCN composites (**a**–**f**) obtained at 293 K.

FT-IR spectra were obtained in order to investigate whether changes in the chemical bonds, after embedding carbon nanotubes (SWCN) in a l,d-PLA matrix, occur. Figure 14 presents the spectra in the whole spectral range between 3100 and 500 cm^−1^.

**Figure 15:**
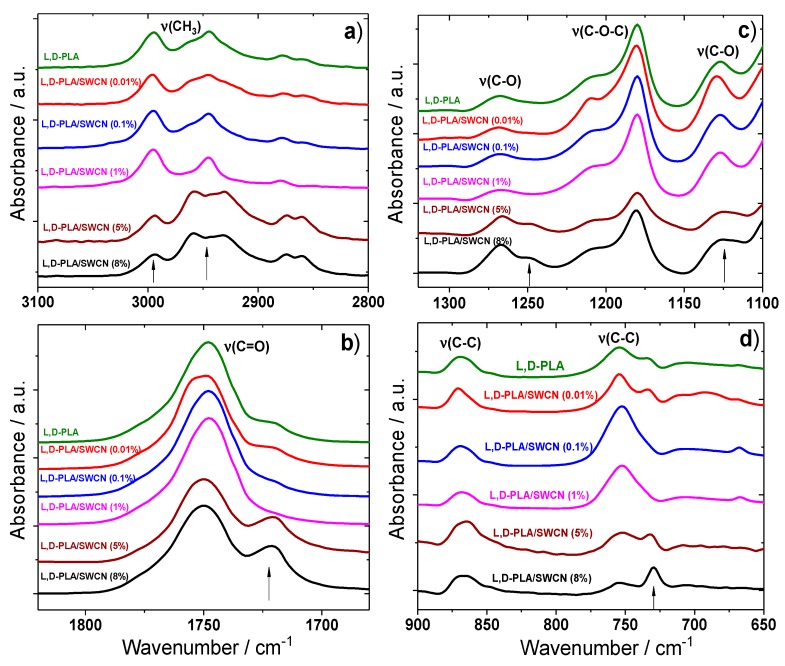
FT-IR spectra of l,d-PLA and l,d-PLA/SWCN composites with different compositions in four different spectra ranges: (**a**) 3100–2800, (**b**) 1820–1680, (**c**) 1320–1100 and (**d**) 900–650 cm^−1^. The bands which change significantly are marked with arrows.

In the spectrum of pure l,d-PLA, the most prominent bands are connected to ν(C–O) and ν(C=O) stretching modes. The first occur in the spectral range of 1300–1000 cm^−1^ and the second one is placed at 1748 cm^−1^. The spectrum also contains stretching and bending vibrations of CH_3_ methyl groups, which occur in the spectral ranges of 3100–2800 and 1480–1300 cm^−1^, respectively. In the low wavenumber range (below 1000 cm^−1^), the ν(C–C) modes are present at 869 and 754 cm^−1^ [55]. The IR spectrum of l,d-PLA fully confirms its proper composition—all the expected essential bands are present in the spectrum. Comparing the results in Figure 14, it can be seen that the spectrum of pure l,d-PLA and the spectra of l,d-PLA/SWCN composites with different SWCN compositions (from 0.01 to 8%) are similar, but some differences are also clearly visible. This means that SWCN moderate l,d-PLA, but do not change the molecular structure of the polymer. The most prominent changes in these spectra can be observed in four spectral ranges (Figure 15), especially for the compositions with 5% and 8% SWCN.

First, in the high-wavenumber range, changes in the bands connected to ν(CH_3_) stretching modes are observed (Figure 15a). The band at 2995 cm^−1^ significantly decreases its intensity in the case of l,d-PLA with 5% and 8% SWCN. The shape of the band with a maximum at 2944 cm^−1^ also changes. It contains three components in pure l,d-PLA: the main one at 2944 cm^−1^ and two shoulders on the sides at 2964 and 2929 cm^−1^. A small amount of SWCN (0.1%) causes the band to become single, but increasing the amount of SWCN to 5% and 8% causes a large increase in the intensities of the two shoulders. Also, the bands at 1748 and 1266 cm^−1^ (ν(C=O) and ν(C–O) modes, respectively) split into two components, and the band at 1127 cm^−1^ (ν(C–O) mode) becomes much broader in the case of the l,d-PLA/SWCN composites with the two highest amounts of SWCN used (Figure 15b,c). Some additional changes can be observed in the low-wavenumber range, where the bands at 754 and 733 cm^−1^ connected with ν(C–C) modes are located (Figure 15d). The band at 733 cm^−1^ decreases in intensity with an increase in the amount of SWCN to 1%. With further increase in the SWCN content, the intensity of this band suddenly increases—for the l,d-PLA/SWCN at 8%, this band becomes more intense than the one at 754 cm^−1^.

In the case of the band at 754 cm^−1^, related to the crystalline region, the opposite situation occurs. Namely, a small amount of SWCN (0.01%) does not significantly change this band compared to pure l,d-PLA. In turn, addition of 0.1% or 1% of SWCN causes a large increase in the intensity of this band. With a further increase in the amount of SWCN (5% and 8%), a sudden drop in intensity is observed. The spectroscopic results indicate that in the case of l,d-PLA/SWCN composites, bonding interactions between the polymer molecules and SWCN occur, and all parts of the polymer are involved. Moreover, from the point of view of the FT-IR method, small amounts of SWCN (up to 1%) moderate l,d-PLA in different ways from larger amounts of SWCN (5% or 8%). The crystallinity of l,d-PLA increases with the addition of SWCN up to 1%, whereas a larger amount of SWCN (5% and 8%) causes its decrease.

### 3.6. Changes in the Chemical Structures of l,d-PLA by SWCN Studied by ^1^H NMR

**Figure 16:**
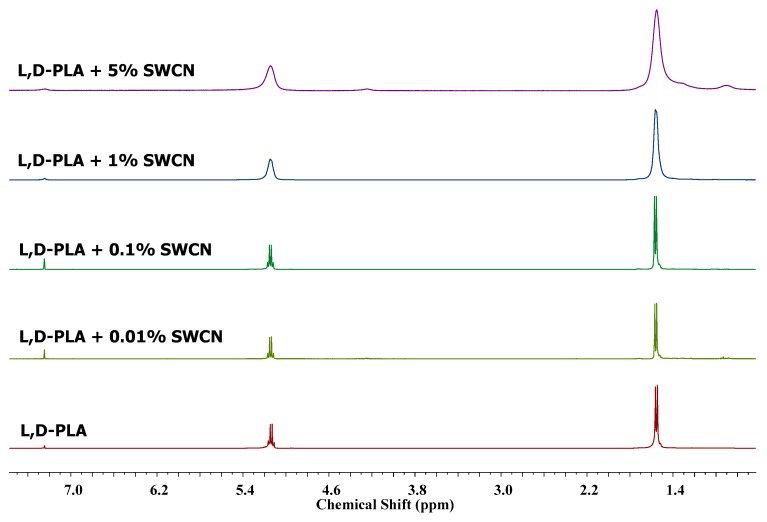
^1^H NMR spectra of pure l,d-PLA and its mixtures with SWCN.

^1^H NMR study was performed on samples containing different amounts of SWCN, in order to detect possible interactions with polymeric matrix. Since the sample are a physical mixture of l,d-PLA and SWCN, no additional signals appeared in the spectra compared to the reference pure l,d-PLA spectrum, as expected. However, the addition of nanomaterials cause broadening of all signals. It was found that the greater the amount of SWCN in sample, the broader the signals (see Figure 16). It seems that carbon nanotubes have an influence on the integrity of the film, and the dissolution process occurs via a swelling due to the possible stabilization of the polymeric matrix with weak interactions.

## 4. Conclusions

As presented in our work, l,d-poly(lactic acid), a known biodegradable polymer, can be used as a flexible and translucent substrate for organic devices, replacing other flexible materials such as PET. Our study showed that a 0.01% SWCN concentration gave translucent supports; however, it did not exhibit sufficient conductivity, which is required for good electric conductors. An increase in the amount of SWCN to 0.1% was accompanied by an increase in conductivity to about 1.01 ×∙10^3^ S/m; however, the films were not fully translucent. Bending of this sample decreased conductivity to 8.51 ×∙10^2^ S/m. The spectroscopic results indicated that in the case of l,d-PLA/SWCN composites at 5% and 8%, bonding interactions between the polymer molecules and SWCN occur, and that all parts of the polymer are involved. It was found that the smallest amount of SWCN, 0.01%, drastically decreases the mechanical properties of thin films, probably due to poor interaction with the polymeric matrix. Meanwhile, the addition of nanotubes from 0.1% to 8% has a smaller impact, and interacts better with the polymer structure. Based on our present results, as well as the discussed literature, the following future goals are proposed:

✓ Optimize technique used for creating hybrid films based on l,d-PLA/SWCN.
✓ Construct organic solar cells using our best l,d-PLA/SWCN films as translucent electrodes.
✓ Introduce l,d-PLA as a flexible, green substrate to build organic solar cells with biodegradable properties.

## Figures and Tables

**Table 1:** Resistivity (*R*), conductivity (σ) and thickness (*d*) of l,d-PLA with selected content of SWCN.

Amount of SWCN	R [Ω] before Deformation	R [Ω] after Deformation	*d* [µm]	*σ* [S/m] before Deformation	*σ* [S/m] after Deformation
0.1%	(2.14 ± 0.45) × 10^3^	(2.50 ± 0.68) × 10^3^	196.9	(5.2± 1.1) × 10^−1^	(4.5 ± 1.2) × 10^−1^
1%	(1.12 ± 0.25) × 10	(1.90 ± 0.33) × 10	207.6	(9.5 ± 2.1) × 10	(5.59 ± 0.98) × 10
5%	1.99 ± 0.22	2.365 ± 0.086	109.6	(1.01 ± 0.11) × 10^3^	(8.51 ± 0.31) × 10^2^
8%	7.3 ± 1.6	8.43 ± 0.85	51.8	(5.8 ± 1.2) × 10^2^	(5.06 ± 0.51) × 10^2^

**Table 2:** Tensile test performance on l,d-PLA with different contents of SWCN.

Composition	Thickness (mm)	Cross-Section Area (mm^2^)	Length (mm)	Stress at Break (MPa)	Elongation at Break (%)
l,d-PLA	0.038	0.38	18.22	71.0 ± 8.9	112.7 ± 25.4
l,d-PLA + 0.01% SWCN	0.145	1.45	19.82	21.8 ± 3.8	104.2 ± 14.0
l,d-PLA + 0.1% SWCN	0.047	0.47	17.86	57.8 ± 5.6	104.4 ± 28.2
l,d-PLA + 1% SWCN	0.054	0.54	17.66	39.4 ± 5.8	103.4 ± 17.4
l,d-PLA + 5% SWCN	0.034	0.34	19.9	45.6 ± 1.5	103.5 ± 7.8
l,d-PLA + 8% SWCN	0.044	0.44	19.08	31.1 ± 8.7	103.4 ± 9.9
